# Experimentelle und numerische Untersuchung von Fluid-Partikel-Interaktionen in Wasser

**DOI:** 10.1007/s00506-023-00960-2

**Published:** 2023-06-07

**Authors:** T. Gold, D. Worf, K. Reiterer, H. Habersack, C. Sindelar

**Affiliations:** Department für Wasser – Atmosphäre – Umwelt, Institut für Wasserbau, Hydraulik und Fließgewässerforschung, Universität für Bodenkultur Wien, Am Brigittenauer Sporn 3, 1200 Wien, Österreich

**Keywords:** tr-3D-PTV-Methode, Amplitudendämpfung, Wirbelablösung, Zusätzliche Masse, Digitale Objektverfolgung, Immersed Boundary Methode, Large Eddy Simulation, tr-3D-PTV Method, Amplitude Decay, Vortex Shedding, Added Mass, Digital Objekt Tracking, Immersed Boundary Method, Large Eddy Simulation

## Abstract

Für die Entwicklung verbesserter Modelle zur Beschreibung des Sedimenttransports in Fließgewässern ist das grundlegende Verständnis der Interaktion zwischen den festen Partikeln und dem bewegten Fluid (Wasser) bedeutend. In diesem Beitrag werden aktuelle Entwicklungen im Bereich der Fluid-Partikel-Interaktion anhand von zwei konkreten Forschungsarbeiten von Gold et al. (2023) und Worf et al. (2022) vorgestellt. Die experimentelle Forschungsarbeit von Gold et al. (2023) untersucht mithilfe von Messmethoden auf dem neuesten Stand der Technik die Strömung rund um Kugeln verschiedener Dichte, die in anfangs ruhendem Wasser oszillieren. Über den gesamten Dichtebereich ($m^{*}=\rho _{S}/\rho _{F}=$ 1,14; 14,95; Dichteverhältnis von Solid und Fluid) konnte für das Kugelpendel eine vergleichbare Charakteristik der Wirbelablösung festgestellt werden. Ebenso wird eine neue Methode der Objektverfolgung (DOT) vorgestellt, welche eine zeitlich und räumlich aufgelöste Analyse der Strömungsstrukturen im Fluidfeld ermöglicht. Gold et al. (2023) zeigen, dass es während der ersten Periode zur Wirbelablösung kommt. Dieser Wirbel pflanzt sich in Richtung Beckenboden fort und dissipiert letztendlich. Weiters wurde ein Dämpfungsoptimum des Kugelpendels im Bereich von $m^{*}=2{,}50$ beobachtet. Außerdem wurde ein Experiment von einem Zylinderpendel mit $m^{*}=4{,}98$ mittels Immersed-Boundary-Methode numerisch untersucht. Dabei wurde der Vorgang der Bildung und Ablösung bis hin zum Zerfall eines Wirbelrings beschrieben. Zusätzlich wurde bei dieser Untersuchung von Worf et al. (2022) die Bildung von Randwirbeln beobachtet, beschrieben und mit der anfänglichen Entwicklung der dreidimensionalen Strömung und „zusätzlichen Masse“ in Verbindung gebracht.

## Abstract (en)

For the development of improved sediment transport models, the basic understanding of the interaction between the solid particle and the moving fluid (water) is important. In this article, current developments in the field of fluid-particle interaction are presented based on two research articles by Gold et al. (2023) and Worf et al. (2022). One presented in this article uses state of the art measurement methods to investigate the flow around spheres of different densities that oscillate in initially resting body of water. For the spherical pendulum a similar vortex shedding characteristic was observed for all investigated fluid density ratios ($m^{*}=\rho _{S}/\rho _{F}=1.14{,}14.95$, density ratio between solid and fluid). A new object tracking method (DOT) is also presented, which enables temporally and spatially resolved analysis of flow structures in the fluid field. The experimental results of Gold et al. (2023) show, that vortex shedding occurs during the first period. This vortex propagates downward and eventually dissipates. Furthermore, a damping optimum of the spherical pendulum in the range of $m^{*}=2.50$ was observed. Additionally, an experiment with a cylindrical pendulum with $m^{*}=4{.}98$ was investigated numerically utilizing an immersed boundary method. The process of creation and separation up to the dissipation of a vortex ring was described. Furthermore, this investigation by Worf et al. (2022) described the creation of tip vortices. These were connected with the development of the three-dimensional flow and added mass coefficient.

## Pendel in der Strömungsmechanik, eine Zeitreise

Die Geschichte von Pendel-Experimenten in der Strömungsmechanik reicht weit zurück. Bereits 1605 bemerkte Galileo Galilei, dass die Periodendauer eines reibungsarmen Pendels konstant bleibt und schaffte somit den Grundstein zur Erfassung der Zeit mittels Pendeluhren. Seitdem beschäftigen sich Forschung, Bildung und Technik mit Pendeln unterschiedlichster Form und Ausführung. Neben der Zeiterfassung sind u. a. ballistische Pendel, Seismometer, Metronome oder gar Massendämpfer in modernsten Hochhäusern anzuführen (Gold et al. 2023; Worf et al. 2022; Mongelli und Battista 2020). Friedrich Wilhelm Bessel entwickelte 1828 das Konzept der zusätzlichen Masse *m*_*a*_, um Pendelschwingungen in Fluiden zu beschreiben. Erfährt ein Körper in einem Fluid eine Beschleunigung oder Verzögerung, so muss er einen Teil des ihn umgebenden Fluidvolumens mitbewegen. Dieses mitbewegte Fluidpaket lässt sich als zusätzlich wirkende Masse des Systems beschreiben. Eine der wohl bekanntesten Meilensteine in der Fluiddynamik bildet die 1851 von George Gabriel Stokes veröffentlichte Arbeit „*On the Effect of the Internal Friction of Fluids on the Motion of Pendulums*“, welche sich ebenfalls mit Pendelschwingungen in Flüssigkeiten und der Beeinflussung durch die Reibungseigenschaften des Fluids beschäftigt (G. G. Stokes 1851). Bis heute stellt das Pendel einen Klassiker in der Schwingungslehre dar, beginnend mit dem gewöhnlichen ungedämpften Pendeln und harmonischen Schwingungen (Mongelli und Battista 2020).

In den letzten Jahrzehnten fand das Pendel im Bereich der Fluiddynamik immer wieder Verwendung zur Untersuchung wirbelinduzierter Schwingungen (Vortex Induced Vibrations – VIV). Hier betrieben Williamson und Govardhan (1997) beziehungsweise Govardhan und Williamson (1997, 2005) umfangreiche Studien unter Zuhilfenahme von Fadenpendeln in stationär gleichförmigen Strömungen. Aus ihren Versuchen ging hervor, dass es bei der Oszillation zu deutlichen Fluktuationen des Strömungswiderstandskoeffizienten sphärischer Körper kommt. Ebenfalls betonen sie die Wichtigkeit einer Visualisierung der vorherrschenden Strömungszustände, um ein besseres Prozessverständnis zu erlangen. Weitere Untersuchungen im Bereich von VIV unter Einsatz modernster Messtechnik wurden von Eshbal et al. (2019) und van Hout et al. (2022) durchgeführt. Diese erweitern die Forschung von Williamson und Govardhan (1997) und Govardhan und Williamson (1997, 2005) und geben Einblick in die vorherrschende Fluid-Partikel-Interaktion, u. a. über Visualisierungen des dreidimensionalen Strömungsfelds.

Weiters setzte sich Mathai et al. (2019) mit der Oszillation von Pendeln im Wasser auseinander. Die durchgeführte Studie beinhaltete Versuche mit Pendelkörpern ohne und unter Auftrieb $(m^{*}=\rho _{S}/\rho _{F}=0{,}33;4{,}98)$. Mithilfe von 2D Particle Image Velocimetry (PIV) erörtern Mathai et al. (2019) die Interaktion zwischen Fluid und Pendel und präsentieren schließlich eine abgeänderte Bewegungsgleichung. Der Fall $m^{*}=4{,}98$ wurde von Worf et al. (2022) aufgegriffen und numerisch untersucht. Aus ihren Simulationen ging hervor, dass von Mathai et al. (2019) festgestellte Änderungen in der zusätzlichen Masse auf ein komplexes 3D-Strömungsfeld zurückzuführen sind. Weiters betonen Worf et al. (2022) die Wichtigkeit einer ganzheitlichen dreidimensionalen Analyse des Strömungsfelds, selbst bei dem in der Literatur oftmals in 2D vereinfachten Fall eines Zylinders. Die gegenständlichen Untersuchungen sollen helfen, die physikalischen Prozesse bezüglich Fluid-Partikel-Interaktionen verschieden geformter Körper besser zu verstehen. Die daraus gewonnen Erkenntnisse sollen in weiterer Folge in der Entwicklung numerischer Modelle bezüglich Sedimenttransport Anwendung finden.

## Versuchsbeschreibung und laser-optische Strömungsmesstechnik

Um die Fluid-Partikel-Interaktion experimentell zu untersuchen, wurde von Gold et al. (2023) eine umfangreiche Messreihe von Pendelschwingungen im anfänglich stehenden Fluid durchgeführt. Die Pendelexperimente fanden in einem 600 mm langen, 300 mm breiten und 300 mm hohen Glasbecken statt. Über dem wassergefüllten Becken wurden mehrere Aluminiumprofile angebracht, welche als Befestigung für Pendel und Auslösemechanismus dienen. In den Experimenten wurden der Kugeldurchmesser $(\mathrm{D}=12{,}7\,\mathrm{mm})$, die Länge des Pendels $\left(\mathrm{L}=200\,\mathrm{mm}\right)$ sowie der anfängliche Auslenkwinkel $(\theta _{0}=37{,}5{^{\circ}})$ konstant gehalten. Über eine Änderung des Fluid-Masse-Verhältnisses $(m^{*}=\rho _{S}/\rho _{F})$ wurden Reynoldszahlen im Bereich von $\mathrm{Re}\ O(10^{4})$ erreicht, wobei acht unterschiedliche Materialien im Dichtebereich $\rho _{S}=(1{,}14;14{,}95)(gcm^{-3})$ verwendet wurden. Neben einer ausführlichen Untersuchung der Schwingungsfrequenz und Amplitudendämpfung für die unterschiedlichen $m^{*}$ stand die Analyse der Wirbelablösecharakteristik im Vordergrund der Studie von Gold et al. (2023). Als Auslösemechanismus diente ein mechanischer Greifer (NIRYO Robotics), welcher über einen Mikrocontroller (OpenCM9.04, Type C) gesteuert wurde. Nach Öffnen des Greifers wird das Messsystem über eine Lichtschranke, welche vom schwingenden Pendel passiert wird, getriggert und die Messung ausgelöst.

**Figure 1:**
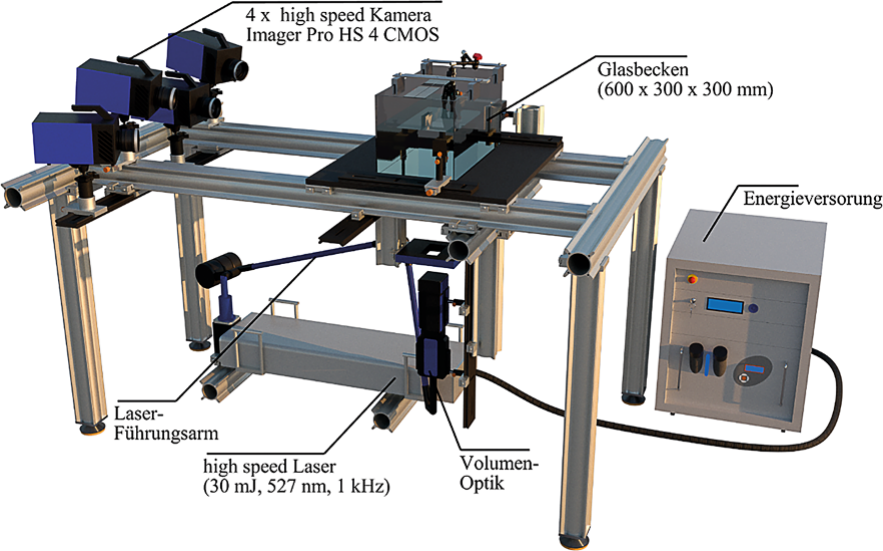

Basis für den beschriebenen Versuchsaufbau sowie das Messequipment bildet hierbei ein Trägersystem aus Aluminium, welches auf schwingungsdämpfenden Nivellierfüßen ruht. Das Herzstück des Messaufbaus bildet ein Highspeed-PTV-System, bestehend aus vier Highspeed-Kameras (Imager Pro HS 4M CMOS) mit einer Auflösung von 2016 × 2016 Pixel. Die Kameras wurden auf zwei verschiedenen Höhenebenen in linearer Konfiguration installiert und jeweils mit einem Scheimpflug-Adapter und einem Zeiss Planar T*85 mm f/1.4 ZE Objektiv ausgestattet. Als Lichtquelle dient ein ND:YLF-PIV-Laser (neodymium-doped yttrium aluminium garnet, diode-pumped, double cavity high-speed laser von Litron LDY series) mit einer Leistung von 30 mJ, einer Wellenlänge von 527 nm und einer Ausgabefrequenz von 1 kHz. Das Laserlicht wird mittels schwenkbarem Führungsarm zur Volumenoptik geführt, welche über zwei optische Linsen den Strahl zu einem Volumen auffächert. Um unscharfe Ränder des aufgefächerten Volumens zu vermeiden, kommt eine mechanische Blende zum Einsatz. Die genaue Anordnung der verwendeten Messtechnik ist in Abb. 1 ersichtlich. Laser und Kamerasystem werden mithilfe einer programmierbaren Timing-Einheit (PTUX von LaVision) synchronisiert und über die Software Davis 10.1 (LaVision) gesteuert.

Die akquirierten Bilder dienen in weiterer Folge als Basis für eine zeitlich aufgelöste 3D Particle Tracking Velocimetry (tr-3D-PTV = time resolved 3D Particle Tracking Velocimetry)-Untersuchung des Strömungsfelds. Heutzutage gilt tr-3D-PTV als eine der fortschrittlichsten Methoden in der laser-optischen Strömungsmesstechnik, welche sich besonders im Feld der Fluiddynamik zur Erfassung und Visualisierung von Wirbelstrukturen eignet (Gold et al. 2023). Neue Entwicklungen im Bereich der berührungslosen laser-optischen Strömungsmesstechnik ermöglichen es, in einem Messvolumen räumlich und zeitlich hochaufgelöste 3D-Fluidgeschwindigkeiten zu ermitteln (Schröder und Schanz 2023; Raffel et al. 2018). Der STB (Shake the Box)-Algorithmus von Schanz et al. (2016), welcher die Auswertezeiten stark verkürzt und dessen Ergebnis räumlich verteilte Partikel-Trajektorien sind, ist eine bedeutende Weiterentwicklung der PTV-Methode. Der VIC+ (Vortex in Cells Method)-Algorithmus von Schneiders und Scarano (2016) nutzt das Ergebnis von STB und führt die Partikel-Trajektorien durch Interpolation auf ein geordnetes kartesisches Raster über. Eine ausführliche Beschreibung dieser Verfahren ist in Gold et al. (2023) zu finden.

## Digitale Objektverfolgung von Wirbelstrukturen (DOT)

Die Untersuchung kohärenter Strömungsstrukturen und Wirbel auf Basis der tr-3D-PTV-Aufnahmen ist oftmals auf eine rein qualitative Beschreibung der Isoflächen-Konturen eines oder mehrerer Zeitschritte beschränkt (Gold et al. 2023). Hierbei gehen allerdings zeitliche und räumliche Informationen verloren, welche in den Aufnahmen enthalten sind. Dennoch haben solch qualitative Beschreibungen Berechtigung, da sie wesentlich zum allgemeinen Prozessverständnis beitragen und zur Validierung numerischer Modelle dienen können (vgl. Zhu et al. 2017; Eshbal et al. 2019). Um die vorliegenden Prozesse noch besser verstehen zu können, wird eine quantitative Analyse der zeitlichen und räumlichen Information durchgeführt. Hierzu wurde von Gold et al. (2023) eine neue Methode vorgestellt, welche die Ermittlung von Trajektorien zusammenhängender Wirbelstrukturen und in weiterer Folge deren Geschwindigkeiten, Bewegungsrichtungen und deren Stabilität im Strömungsfeld, basierend auf der visuellen Darstellung (z. B. Isoflächen), ermöglicht.

In diesem Beitrag wurden zusammenhängende Strukturen mithilfe von Isoflächen der Wirbelgröße (ω) und mittels Q‑Kriterium visualisiert. Beim Q‑Kriterium werden Wirbelstrukturen als Regionen definiert, in welchen das Maß der Rotation dem Maß der Scherung überwiegt (Hunt et al. 1988).

1 $$Q=1/2(\parallel \Omega \parallel ^{2})-(\parallel S\parallel ^{2})$$

**Figure 2:**
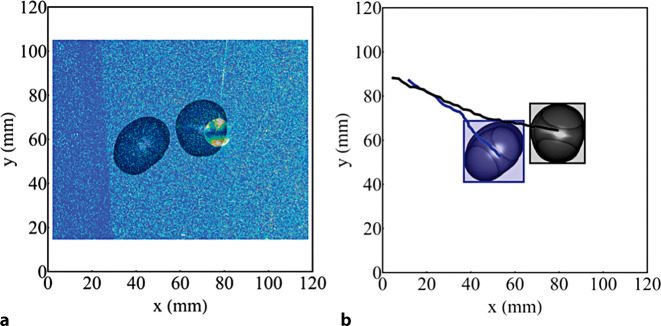

**Figure 3:**
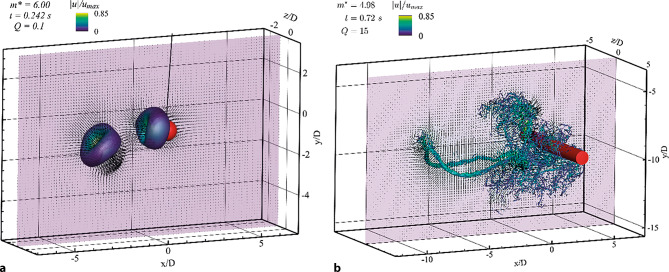

Hierbei bildet *Ω* den antisymmetrischen und *S* den symmetrischen Anteil des Geschwindigkeitsgradienten. Folglich zeigen Bereiche mit $Q > 0$ Wirbelstrukturen im Strömungsfeld an. Entsprechende Wirbelstrukturen sind in Abb. 2 anhand der Wirbelgröße (ω) und in Abb. 3 anhand des Q‑Kriteriums dargestellt.

Die ermittelten Isoflächen dienen anschließend als Grundlage für die DOT-Methode von Gold et al. (2023). Für einen Versuch mit einem bestimmten $m^{*}$ wurde jeweils ein konstanter *ω*-Wert gewählt. Um diesen Wert nicht beliebig zu wählen, sondern um einen möglichst objektiven Vergleich zwischen Pendelversuchen mit unterschiedlichem $m^{*}$ zu gewährleisten, wurde eine Skalierung der Flächen anhand der Periodendauer während des ersten Schwingvorgangs angewendet. Aus der experimentell ermittelten Periodendauer ergibt sich die empirische Skalierungsfunktion $\left| \omega \right| =16/T$. Die weitere Auswertung erfolgte in Wolfram Mathematica 12. Nach Import der Bilder wurde als erster Schritt eine semantische Segmentierung, basierend auf einer Binarisierung und anschließender Farbumkehr, durchgeführt. Je nach Intensität der jeweiligen Pixel führt dies zu Bildern mit „Ones“ (schwarz) und „Zeros“ (weiß). Im Anschluss wurden für die jeweiligen Zeitschritte Begrenzungsrahmen für zusammenhängende Wirbelstrukturen, welche mehr als 2000 Pixel umfassen, bestimmt. Die anhand der Rahmenkoordinaten ermittelbaren Trajektorien geben, zusammen mit der zeitlichen Information der Bilder, Aufschluss über die Ausbreitungsgeschwindigkeiten der Wirbelstrukturen. Abb. 2 zeigt die beispielhafte Auswertung mittels DOT-Methode inklusive Originalbild, Isoflächen, Begrenzungsrahmen und Trajektorien.

## Methoden der numerischen Untersuchung

Ein Experiment ($m^{*}=4{,}98$) von Mathai et al. (2019) und ein Experiment ($m^{*}=2{,}50$) von Gold et al. (2023) wurden mit dem Virtual Flow Simulator (VFS-Geophysics)-Modell mit Large Eddy Simulation numerisch simuliert. VFS-Geophysics nutzt dazu eine Form der Immersed-Boundary-Methode (IBM), dazu wird die Geometrie mittels Oberflächennetzen, sogenannten Immersed Boundaries (IB), dargestellt und die Fluidbewegung auf einem strukturierten nichtkonformen Rechengitter berechnet. Die verwendete IBM heißt Curvilinear Immersed Boundary (CURVIB)-Methode (Ge und Sotiropoulos 2007), welche krummlinige Rechengitter erlaubt und zur Berechnung die Zellen des Rechengitters in drei Kategorien unterteilt: Fluid‑, Solid- und IB-Zellen. Die (gefilterten) Navier-Stokes-Gleichungen werden nur auf den Fluidzellen gelöst, während auf den IB-Zellen die Geschwindigkeit mittels einer wandnormalen Interpolation berechnet wird, damit die Haftbedingung an der IB erfüllt ist.

Im Fall von bewegten Objekten haben IBMs den wesentlichen Vorteil, dass sie keine ressourcenintensiven Änderungen am Rechengitter vornehmen müssen. Für die Bewegung des Pendels werden die Newtonschen Bewegungsgleichungen mittels kinematischer und dynamischer Randbedingungen an die Berechnung der Fluidbewegungen gekoppelt. Dazu werden im Fall von VFS-Geophysics die auf das Objekt wirkenden hydrodynamischen Kräfte auf den nächstgelegenen Fluidzellen des Rechengitters berechnet. Für die simulierten Experimente konnte aufgrund des Verhältnisses zwischen zusätzlicher und tatsächlicher Masse des Pendels für die Berechnung der Pendelbewegung ein explizites Lösungsverfahren, welches ohne Neuberechnung der Fluidbewegung auskommt, verwendet werden, ein sogenanntes Loose Coupling (Borazjani et al. 2008). Eine ausführliche Beschreibung aller verwendeten numerischen Methoden findet sich in Worf et al. (2022).

## Charakterisierung der Wirbelablösung

Zur Charakterisierung der Wirbelbildung werden hier die experimentellen Ergebnisse des Kugelpendels von Gold et al. (2023) mit den numerischen Ergebnissen des Zylinderpendels von Worf et al. (2022) verglichen.

Für alle von Gold et al. (2023) untersuchten Materialien erzeugte die Bewegung der Kugel am Beginn der Schwingung eine torusartige Wirbelstruktur. Der Durchmesser dieser Wirbelstruktur *D*_*vor*_ betrug für alle $m^{*}$ ungefähr 2*D*, wenn der Grenzwert der Wirbelintensität-Isoflächen mit der Skalierungsfunktion $\left| \omega \right| =16/T$ bestimmt wurde. Im weiteren Verlauf beginnt sich diese Struktur in zwei eigenständige Wirbel gleicher Größe zu trennen. Einer der Wirbel löst sich in weiterer Folge ab, dargestellt in Abb. 3a, und bewegt sich in einer Neigung, ähnlich der Anfangsauslenkung *θ*_0_, nach unten. Während der Abwärtsbewegung bleibt der Ring bemerkenswert stabil, bis er nach Erreichen seiner Endgeschwindigkeit langsam dissipiert. Eine ähnliche Wirbelstruktur wurde experimentell von Mathai et al. (2019) und numerisch von Worf et al. (2022) für Zylinderpendel festgestellt, diese ist in Abb. 3b dargestellt. Aufgrund der unterschiedlichen Geometrie des Pendels ist der gebildete Vortexring beim Zylinder größer und die Entwicklung des Wirbels ist, aufgrund des Übergangs zwischen hauptsächlich dreidimensionaler Strömung am Rand des Zylinders zu hauptsächlich zweidimensionaler Strömung in der Mitte des Zylinders, in mehreren Stadien zu beschreiben. Diese Stadien lassen sich aus Sicht der 2D-Strömung über die kurzfristige Bildung einer Kármánschen Wirbelstraße erklären. Bei dem gewählten Experiment verbinden sich der erste und vierte abgelöste Wirbel im Bereich des Zylinderrands zu einem Ring, der sich aufgrund des vom Pendel auf das Fluid übertragenen Impulses abwärts bewegt. Dabei ist der vierte abgelöste Wirbel aufgrund der hohen Reynoldszahl ($Re=33700$) am Beginn nicht als solcher zu erkennen, sondern bildet sich aus einer turbulenten Region hinter dem Zylinder im Lauf der Entwicklung des Vortexrings. Die Lebensdauer des Wirbelrings ist länger als es dauert, bis der Ring den Boden des Behälters erreicht. Dort wird der Ring durch das vom Boden abgelenkte Fluid nach außen gedrückt und der Radius des Rings vergrößert sich, bis der äußerste Teil des Rings die seitlichen Wände erreicht. Dieser Teil wird dadurch nach oben abgelenkt. In Folge der durch die unterschiedlichen Bewegungsrichtungen ausgelösten Streckung zerfällt der Ring kurz danach.

Im Gegensatz zum Kugelpendel, bei dem sich keine Wirbelstraße ausbildet, bilden beim Zylinderpendel, zusätzlich zum abwärts wandernden Vortexring, der zweite und dritte abgelöste Wirbel ein Paar, welches sich aufwärtsbewegt. Aufgrund der großen Reynoldszahl und damit verbundenen Turbulenz zerfällt das Wirbelpaar sehr schnell in eine turbulente Region. Ähnliche Bewegungen der abgelösten Wirbel konnten von Mongelli und Battista (2020) auch in einigen Fällen ihrer 2D-Simulationen beobachtet werden.

**Figure 4:**
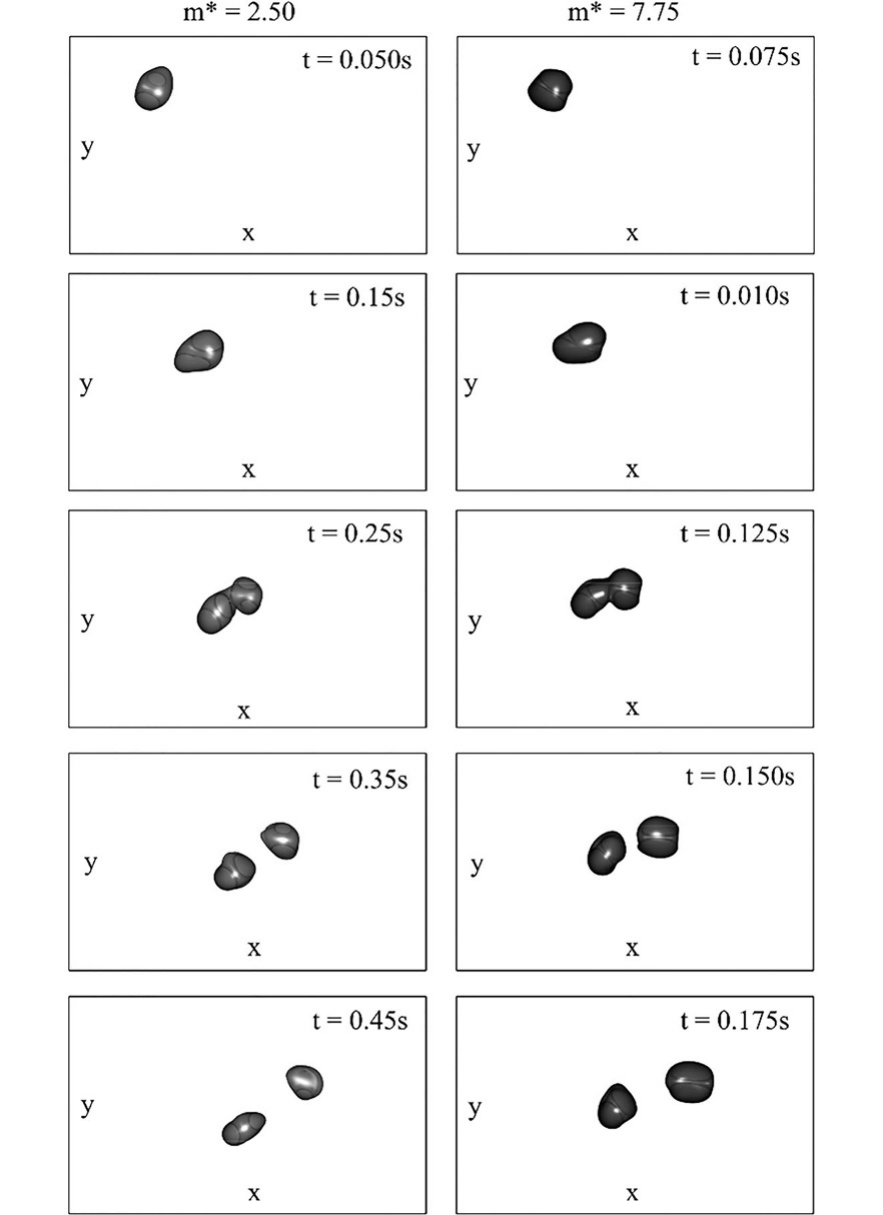

Der zweite, von Gold et al. (2023) beobachtete, Wirbel blieb vorerst, wie in Abb. 3a für $m^{*}=6{,}00$ dargestellt, auf der Pendeltrajektorie. Der Vorgang der Wirbelablösung zeigte sich unabhängig von der Dichte der Kugel und ist in Abb. 4 für die Masseverhältnisse $m^{*}=2{,}50$ und $m^{*}=7{,}75$ anhand von jeweils fünf Zeitschritten dargestellt. Startzeitpunkt und Zeitschritt wurden dabei so gewählt, dass die Position der Wirbelstrukturen für beide Dichteverhältnisse möglichst synchron bleibt.

Mittels der DOT-Methode von Gold et al. (2023) konnte der exakte Zeitpunkt der Wirbelablösung *t*_*vs*_ bestimmt werden. Einen theoretischen Ansatz zur Bestimmung der Wirbelablösefrequenz bietet die Strouhal-Zahl $S_{r}=f_{vs}D/v_{p}$. Diese setzt somit den Zeitmaßstab der Wirbelablösefrequenz *f*_*vs*_ und den Zeitmaßstab der Fluidgeschwindigkeit (relative Pendelgeschwindigkeit *v*_*p*_) über eine charakteristische Länge (hier Kugeldurchmesser *D*) in Relation. Für die hier betrachteten Reynoldszahlen und Kugeln lässt sich die Strouhal-Zahl konstant als $S_{r}=0{,}21$ annehmen. Dadurch ergibt sich die Formel für den Zeitpunkt der ersten Wirbelablösung als Gl. 2.

2 $$t_{vs}=\frac{1}{f_{vs}}=\frac{D}{S_{r}v_{p}}$$

**Figure 5:**
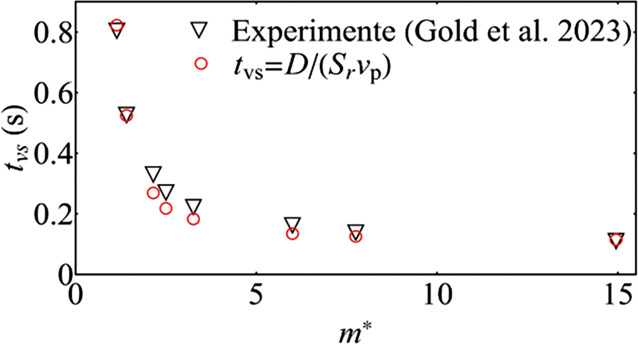

Ein Vergleich zwischen gemessenem und errechnetem Zeitpunkt der ersten Wirbelablösung *t*_*vs*_ ist in Abb. 5 dargestellt. Hier ist für alle betrachteten Dichteverhältnisse von Kugel und Fluid eine sehr gute Übereinstimmung zwischen theoretischem Ansatz und Experiment zu erkennen.

**Figure 6:**
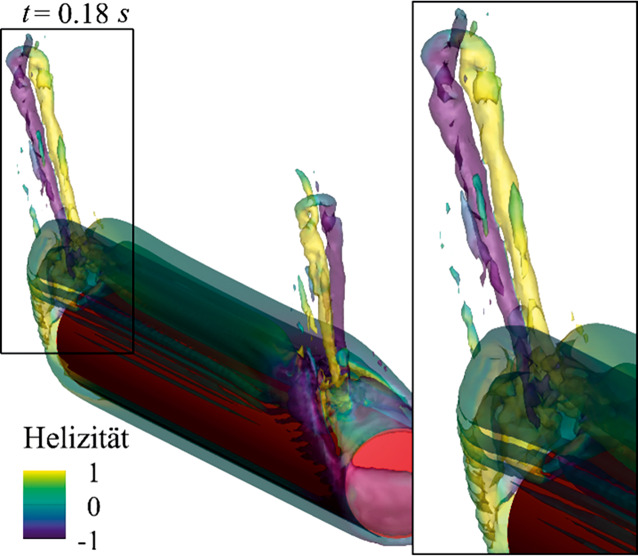

Im Fall des Zylinderpendels entwickeln sich zusätzlich zur Wirbelablösung am Beginn der Bewegung an den Enden des Zylinders Randwirbel, welche aufgrund des dreidimensionalen Verhaltens des Kugelpendels nur beim Zylinder vorkommen. In Abb. 6 sind diese Randwirbel dargestellt und mit der Helizität eingefärbt, was die Drehrichtung der dargestellten Wirbel anzeigt. Es zeigen sich je zwei gegenläufige Wirbel an beiden Enden des Zylinders. Diese hatten eine Lebensdauer von zirka 0,24 *s*. Anhand der berechneten Kräfte konnte von Worf et al. (2022) gezeigt werden, dass sich während desselben Zeitrahmens der Koeffizient der zusätzlichen Masse *m*_*a*_ vom theoretischen Wert für „unendlich lange“ Zylinder von 1 auf den von Mathai et al. (2019) gemessenen Wert von 0,53 annähernd linear reduziert. Das zeigt einen Zusammenhang zwischen den Randwirbeln mit der Entwicklung der 3D-Strömung.

## Schwingungsfrequenz und Amplitudendämpfung

Tab. 1 fasst die gemessenen Periodendauern des ersten Schwingungszyklus *T* und die maximalen Auslenkungen am Ende der ersten Schwingung *θ*_*max*_ zusammen. Die Periode *T* nimmt nicht-linear mit zunehmendem Masseverhältnis $m^{*}$ ab, während *θ*_*max*_ am Ende des ersten Schwungs logarithmisch mit zunehmenden $m^{*}$ ansteigt.

**Table 1:** 

$m^{*}$	*T* (s)	*θ*_*max*_ (rad)
1,14	4,75	0,048
1,41	2,70	0,067
2,15	1,60	0,107
2,50	1,40	0,133
3,26	1,30	0,156
6,00	1,10	0,269
7,75	1,05	0,330
14,95	0,95	0,440

**Figure 7:**
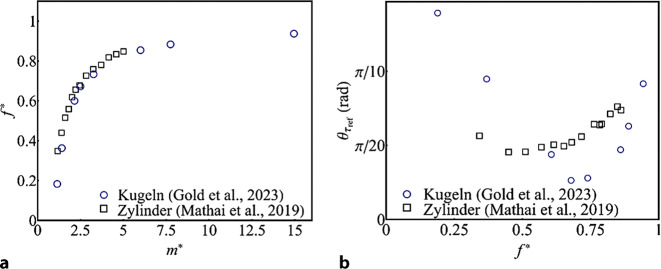

Die Schwingfrequenz des Pendels $f=1/T$ lässt sich mit Eigenfrequenz des Pendels $f_{n}=\left(1/2\pi \right)\sqrt{g/L}$ normalisieren (Gold et al. 2023). Abb. 7a zeigt diese normalisierte Frequenz $f^{*}=f/f_{n}$ für Kugeln und Zylinder mit verschiedenen Masseverhältnissen $m^{*}$. Sowohl für Kugeln als auch für Zylinder nimmt die Abweichung zwischen Eigenfrequenz *f*_*n*_ und gemessener Frequenz *f* mit abnehmendem $m^{*}$ deutlich zu. Durch das Anpassen einer Umhüllenden an den zeitlichen Amplitudenverlauf lässt die Amplitudenabnahme $\theta _{{\tau _{\mathrm{ref}}}}$ zum Zeitpunkt $\tau _{\mathrm{ref}}=3\pi \sqrt{L/g}$ bestimmen. Durch die Gegenüberstellung von $\theta _{{\tau _{\mathrm{ref}}}}$ und $f^{*}$ in Abb. 7b ergibt sich für Kugeln ein Dämpfungsoptimum für $f^{*}\approx 0{,}7$, was einem Masseverhältnis von $m^{*}\approx 2{,}5$ entspricht. Für Zylinder liegt das Optimum bei $m^{*}\approx 2{,}0$. Dieser Unterschied zwischen den beiden Formen lässt sich mit der unterschiedlichen Trägheit, ausgedrückt durch den Koeffizienten der „virtuellen oder zusätzlichen Masse“ *m*_*a*_, erklären. Der entsprechende Massekoeffizient *m*_*a*_ ist für „unendlich lange“ Zylinder 1 und für Kugeln 0,5. Hinsichtlich $m^{*}$ zeigen beide Formen einen nicht-monotonen Dämpfungsverlauf, der für Kugeln jedoch stärker ausgeprägt ist (vgl. Abb. 7b). Zum Teil lässt sich dies mit dem Einfluss des nicht-linearen Strömungswiderstands erklären. Der Strömungswiderstandsbeiwert *c*_*w*_ für Kugeln ist für die hier gegenständlichen Reynolds-Zahlen *Re* generell kleiner als für Zylinder. Dies zeigt sich in der langsameren Amplitudendämpfung der Kugeln. Weiters haben wirbelinduzierte Schwingungen großen Einfluss auf den Strömungswiderstand, weshalb die Kenntnis der Wirbel-Ablösecharakteristik von großer Bedeutung für die Untersuchung von Fluid-Struktur-Interaktionen ist.

Der Kenntnis der Dämpfungseigenschaften kommt in der Konstruktion mechanischer Systeme große Bedeutung zu. Die vorliegenden Zusammenhänge können zum Beispiel in der Gestaltung von Schiffskranen Anwendung finden.

## Zusammenfassung und Schlussfolgerung

Dieser Artikel präsentiert eine detaillierte Untersuchung der Fluid-Struktur-Interaktionen anhand von Pendel-Experimenten unter Wasser für eine große Bandbreite von Dichteverhältnissen $m^{*}$. Die Anwendung moderner laser-optischer Strömungsmesstechnik (tr-3D-PTV), Analysemethoden (STB and VIC) und digitaler Objektverfolgung (DOT) ermöglichte die Beschreibung eines charakteristischen Wirbelablöseverhaltens. Dieses ist beim Kugelpendel von der Ausbildung eines torusförmigen Wirbelrings, der sich später in zwei gleich große Wirbelringe auflöst, geprägt. Einer dieser Wirbelringe bleibt auf der Kugelbahn, während sich der zweite ablöst und, auf einer annährend linearen Bahn, nach unten wandert. Hierbei ergab sich für alle $m^{*}$ ein annährend gleicher Winkel. Eine Analogie der Wirbelgröße $D_{\mathrm{vor}}\approx 2D$ ergibt sich für alle $m^{*}$, wenn die Wirbelstärke mit der Periodendauer *T* skaliert wird. Ein Vergleich des mit der DOT-Methode von Gold et al. (2023) bestimmten Zeitpunkts der ersten Wirbelablösung und dem theoretischen Ansatz der Wirbelablösefrequenz über die Strouhal-Zahl zeigte eine sehr gute Übereinstimmung. Dies bestätigt die Verlässlichkeit der DOT-Methode von Gold et al. (2023). Die Entwicklung des abgelösten Wirbelrings wurde mit der numerischen Simulation mit IBM eines Zylinderpendels von Worf et al. (2022) verglichen und im Zuge dessen genauer betrachtet. Dazu wurde die Entstehung aus einer Kármánschen Wirbelstraße beschrieben. Anstelle der zwei abgelösten Wirbelringe beim Kugelpendel entsteht beim Zylinder ein großräumiger, zusammenhängender Wirbelring. Zusätzlich wurde die Entwicklung der 3D-Strömung zu Beginn der Zylinderpendelbewegung mit Randwirbeln visualisiert und in Zusammenhang mit der anfänglichen Reduktion der zusätzlichen Masse gebracht. Weiters lässt die Analyse der Schwingfrequenz und Amplituden ein nicht-monotones Dämpfungsverhalten sowohl für Kugeln als auch Zylinder erkennen. Für Kugeln findet sich ein Dämpfungsoptimum bei $m^{*}\approx 2{,}5$, für Zylinder bei $m^{*}\approx 2{,}0$. Diese Informationen hinsichtlich masseabhängiger Strukturantwort und der zugehörigen Wirbelablösecharakteristik kann für die Entwicklung maritimer Infrastruktur und die Stabilität von Schiffskranen nützlich sein. Weiters sollen die Erkenntnisse zur Fluid-Partikel-Interaktion wie Wirbeldynamik und „zusätzlichen Masse“ in die Entwicklung numerischer Sedimenttransport- und Partikel-Kollisionsmodelle einfließen.
